# The two sides of the scalpel: The polarizing image of surgery in early cinema

**DOI:** 10.1371/journal.pone.0279422

**Published:** 2022-12-21

**Authors:** Dennis Henkel

**Affiliations:** Faculty of Medicine and University Hospital Cologne, Institute for the History of Medicine and Medical Ethics, Cologne University, Cologne, Germany; Aminu Kano Teaching Hospital, NIGERIA

## Abstract

This paper uses extensive database research, film viewing and literature review to show how the field of surgery was staged in the early days of film history. It can be shown that–although surgical medicine was a subject in transition, and many scientific breakthroughs (anesthesia und antisepsis) made surgery less painful and more complication-free–filmmakers still frequently resorted to horror memories of the past and created a questionable, or ambivalent image of the surgeon, sometimes as extreme as the “lunatic with a scalpel” stereotype, blurring the line between genius and madness. But there were also positive staging’s: The surgical intervention was often captured on the screen as a last resort for clinically hopeless cases, with the surgeon often presented as a “deus ex machina”, the savior out of nowhere. Other specialties, however, such as plastic surgery, were mostly positively dramatized, which reveals a stark contrast to research about the representation of the field in the sound film era. A view at the fields of neurosurgery and (selectively) opthalmo-surgery rounds out the panorama of forty-one surgical films. In summary, it is shown that the early surgical film depicts the specialty and the surgeon in a highly ambivalent way, from savior to monster thereby reflecting one of the most significant transitions in the history of surgery and showing us what image was presented to the public–and thus to potential patients–in the movie theaters.

## Introduction

Medicine and doctors have played a major role in cinema from the beginning of film history [1]. Filmmakers have preferred neuropsychiatric disorders because they appear perfectly suitable for arousing fear and strong emotions in the audience [2, 3]. However, the frequency of displaying various pathologies on-screen has increased, while simultaneously arousing scientific interest from physicians and film scientists [4–10]. One missing piece is the portrayal of surgery in early cinema. The period around 1900 was a thrilling one for surgery. The standardization of antisepsis and increasingly effective anesthesia allowed the field to celebrate one success after another. Great physicians such as Theodor Billroth, Ferdinand Sauerbruch and Rudolf Nissen became dazzling celebrities. However, if one looks at the cinematic representation of the field at the time, a strange connotation glimmers through, questioning the image of the omnipotent savior: An uneasiness accompanies the camera’s point of view, seemingly skeptically capturing the medical superstars, often staged in sinister plots as terrifying figures. How do we explain this contradiction? Are the groundbreaking successes of surgery unrecognized? To sharpen our understanding of the public’s perception of physicians and medicine in general, then and now, we need to analyze how the field is portrayed in mass media. This study builds on the vast body of research on the depiction of medicine in films by historians of medicine and general film studies (important works are referenced to in the course of this paper) and closes an important gap in medicine history: The depiction of the first portrayals of surgery in the history of cinema and the discovery of important historical sources for medical historians.

## Materials and methods

The research strategy can be divided into identification, acquisition, and analysis. The keywords "surgeon," "surgery" and "operation" were entered while searching movie databases [11–13] followed by interdisciplinary literature research and film viewing. A relevance test of the content led to the exclusion of productions in which the surgical field was not present in an important manner the plot or its drama. This tedious task was followed by an autoptic examination of the material. The exclusion criteria were as follows: rhetorical mention of the field only, even if repetitive, educational films, documentaries, and patient videos. Thus, this paper is restricted to fictional films produced for cinema intended for a broad audience. Furthermore, the selection was focused on the early cinema before 1930, the transition from silent film to sound film. This is not only because the sound film era is well covered in scientific research [7, 28, 32], but also because it is a particularly interesting period of time for the history of cinema: Movies were still almost free from censorship (the Hayes Code–the first industry guidelines for self-censorship–was not implemented until the early 1930s) and provided an authentic picture of the representation of medicine.

## Results and discussion

When cinema first came into being, it had its own characteristics, which are often unknown to the modern viewer and are therefore presented in a very brief overview: The first films were attractions shown at fairgrounds and mostly short films of a few minutes in duration. The plots were rarely constructed as more than a stunning or amusing snapshot, but soon the initial amazement at the technical wonders of moving pictures faded. Consequently, the plots became more complex, and the running time expanded. This process evolved over at least two decades, until the feature-length film overtook the short films as the standard we all know today. Films from this period, however, are in a desolate state of preservation (this circumstance explains why only films from industrialized countries like USA, Germany and France are preserved and represented in this paper) and most of them are considered lost–like the first surgery film in cinema history: *Chirurgien américain* (France 1897, Georges Méliès) [14]. Méliès was one of the first influential filmmakers to emerge from the early stages of cinema and is still known for classics like *Le Voyage dans la Lune* (France, 1902). *Chirurgien américain* utilizes the special effects–typical of Méliès style–to visualize a transplant of both legs to a tramp. In the following, the patient must be satisfied with more procedures, because aesthetic results do not appeal to him. Accordingly, the first representation of plastic surgery can be found as well, when the patient’s head is miraculously replaced by a more attractive one [15]. The first female director of "surgical films", Alice Guy, created a similar work with *Surgery fin de siècle* (France 1900). Today, Alice Guy is considered the first significant female filmmaker in cinema history with a career spanning several decades [16]. Her *Surgery fin de siècle*–fortunately, preserved–shows an operating room where a patient’s limbs are amputated and resewn. One can already anticipate the feelings the film is going to evoke as, apart from the marvelous special effects, the "Please do not cry" messages adorning the theatre walls–an ironic jab that accentuates the severe pain related to surgical procedures, stares down at the viewer. Considering that anesthesia was already established at that time, this reference can be considered misinformation [17].

Film example 1: *Une indigestion* (France 1902, Georges Méliès) takes us back to the special effects films of the Houdini admirer Méliès. Cinematically, the director presents more along the lines of the works already mentioned, the immediate operation: A patient with severe abdominal pain comes to a doctor’s office, where the resourceful surgeon immediately reaches for the scalpel. Following this familiar pattern, the limbs are removed, and the surgeon opens the abdominal cavity and removes the most unexpected equipment such as forks and lamps. As these procedures failed to provide any proper pain relief, the Medicus cuts off the patient’s head, fills his torso with the assistance of a futuristic water pump and miraculously reassembles the patient. The absurd operation is a success, and the client willingly pays the doctor’s fee [18]. Méliès seems to have been inspired by an 1892 theatrical performance that lured audiences to the box office with optical illusions called "Le Charlatan Fin de Siècle" [19] The popularity of grotesque-utopian operations was therefore not solely due to novel cinematic trick effects. This is further illustrated by headlines from the German press of the time, which wrote skeptically about the hemicraniectomies performed by the French brain surgeon Eugène-Louis Doyen: The surgical instruments would look like a "…pincher, a "mortise and tenon machine" and a pipe saw" [20]. The first representations of surgery in film were thus more than mere displays of trick effects; the motion pictures reflected the circumstances and prejudices of the time with which surgeons had to cope.

The short film was not completely replaced by the feature-length film; for a long time, short animated, slapstick, or experimental films were shown as openers to feature-length films, where the surgical theme reappeared. Examples would be the animated *I’m Insured* (USA 1916, Harry Palmer), in which a man tries to provoke an accident in order to get insurance money. But he is quickly confronted with the reality of being a traumatized surgery patient. In *No Noise* (USA 1923, Robert F. McGowanin), the "Little Rascals" turn a surgical ward into mayhem after one of them had his tonsils removed. They try to stay mainly because they get plenty of ice cream after the procedure. Following this, the gang wreaks havoc on X-ray machines, and exhibition skeletons and is having questionable fun with laughing gas, until they find themselves with disturbing complications (erysipelas, amputated toes, etc.). Medically enlightened, they flee in agony. Also worth mentioning is the frequently used cliché of the "blood-splattered surgeon", which was promptly used to induce moments of sheer horror. A well-known example would be the butcher-like surgeon in *Good Night*, *Nurse* (USA 1918, Roscoe Arbuckle), portrayed by the famous slapstick-comedian Buster Keaton.

### The surgeon as an omnipotent healer

It did not take long for the feature-length film present the surgeon-stereotype of "the savior with the scalpel", as displayed in *Das Tagebuch des Dr*. *Hart* (Germany 1916, Paul Leni), showing the routines of military physicians: Dr. Hart is ordered to the front. As soon as he arrives, we see the physician disinfecting wounds, giving first aid, administering injections, and rescuing the critically wounded by surgical intervention (which is not shown in detail). The heroic deeds are embedded in a rather tedious war propaganda film, which is in line with the intentions of the filmmakers [21]. In this debut work, director Paul Leni shows himself artistically well below the standards of his later works, primarily the expressionistic *Das Wachsfigenkabinett* (Germany 1924), but created the first undoubtedly positively connoted, almost heroic surgeon figure in cinema history.

Two films starring Hollywood darling Mary Pickford stylize the surgeon as a destiny-defining savior. *Stella Maris* (USA 1918, Marshall Neilan) tells the story of a paraplegic young woman and her faithful companion, a dog. Despite her fate, she displays an irrevocably naïve optimism. This positive attitude towards life is indirectly rewarded when a surgeon suddenly appears and restores Stella’s ability to walk.

Film example 2: *Pollyanna* (USA 1920, Paul Powell) can almost be considered a version of Stella Maris, as the similarities in the storyline illustrate: Again, Mary Pickford embodies a naïve and thoroughly optimistic girl, which imposes like a euphemism. She tries to animate everyone around her to play "the glad game", a game in which one simply mentions the things that make oneself happy. The girl even uses her spare time to cheer up sick neighbors in agony with her altruistic spirit–early Hollywood kitsch! But destiny knocks on her door, when a car accident leaves her in a wheelchair–but Pollyanna remains true to her philanthropic and optimistic nature. A certain Dr. Chiltern–renowned neurosurgeon–was a friend of the family, but an argument with Pollyanna’s aunt ended their relationship. However, as he seems to be the only one capable of performing the risky operation, the resentful aunt reconciles with the surgeon. Now the path is set for the happy ending: the operation succeeds, and Pollyanna can slowly take her first, shaky steps out of her wheelchair.

Both Pickford films use the surgeon as a deus ex machina, a dramatical figure, who suddenly appears and only serves the function of inducing the happy end (with his surgical skills). However, the moment of faith is–as in Stella Maris–the driving force behind the heartwarming finale and makes the film a work of religious-Christian virtues, in which the physician only serves as a “reward” for her irrefutable sense of justice and charity.

Other examples of life-altering surgical interventions come from the field of ophthalmological surgery. In the lost *When Love Was Blind* (USA 1911, Lucius J. Henderson), a blind woman is given her sight back by a surgeon. But when the patient prematurely removes her bandages, she loses her eyesight again and must accept her “dark” destiny. In the identically titled motion picture from 1917 (USA, Frederick Sullivan), the operations restore the patient’s vision without any complicating setbacks [22]. Another notable case is *Back Pay* (USA 1922, Frank Borzage): a veteran lost his eyesight during the war, becomes delirious and ultimately dies from a "gassed left lung". Here, the professional and competent physician is more than willing to help, but all efforts are in vain; the palliative situation cannot be prevented, and the surgeon fails–but without being displayed in a judgmental way.

Far more often, however, the surgeon figure was used by filmmakers to generate horror, shivers and fear for the audience, which will be analyzed in more detail in the following paragraph.

### The surgeon as a monster

The range of questionable characters among the screen surgeons is wide. Perhaps the least despicable of these character types is the heartless, money driven “Raffke”, who requests huge amounts of money for their services. The "unaffordable operation" often functions in films as a pretext for an indirect defamation of the medical profession, deeming it greedy, but sometimes also to voice social criticism–nevertheless, this kind of portrayal leaves a negative impression of surgical care. An example of this would be *The Affairs of Anatol* (USA 1921, Cecil B. DeMille) by the famous director DeMille, who is remembered by cineastes for films like *The Ten Commandments* (USA 1956), which enriches the television program every Easter. In DeMille’s silent film we meet Anatole, the wife of a severely injured war veteran, who must raise $3,000 to enable her husband undergo the lifesaving operation. Thanks to shady jobs, she raises the money, but surgery cannot save her husband, as a phone call with the surgeon reveals.

A similar set-up appears in *Schmutziges Geld* (Germany, and Great Britain 1928, Richard Eichberg), which also deals with the torments of a young woman who has to raise money for the operation of her blinded boyfriend. The attempt has a futile outcome for the desperate woman and the operation never materializes [23].

**Fig 1:**
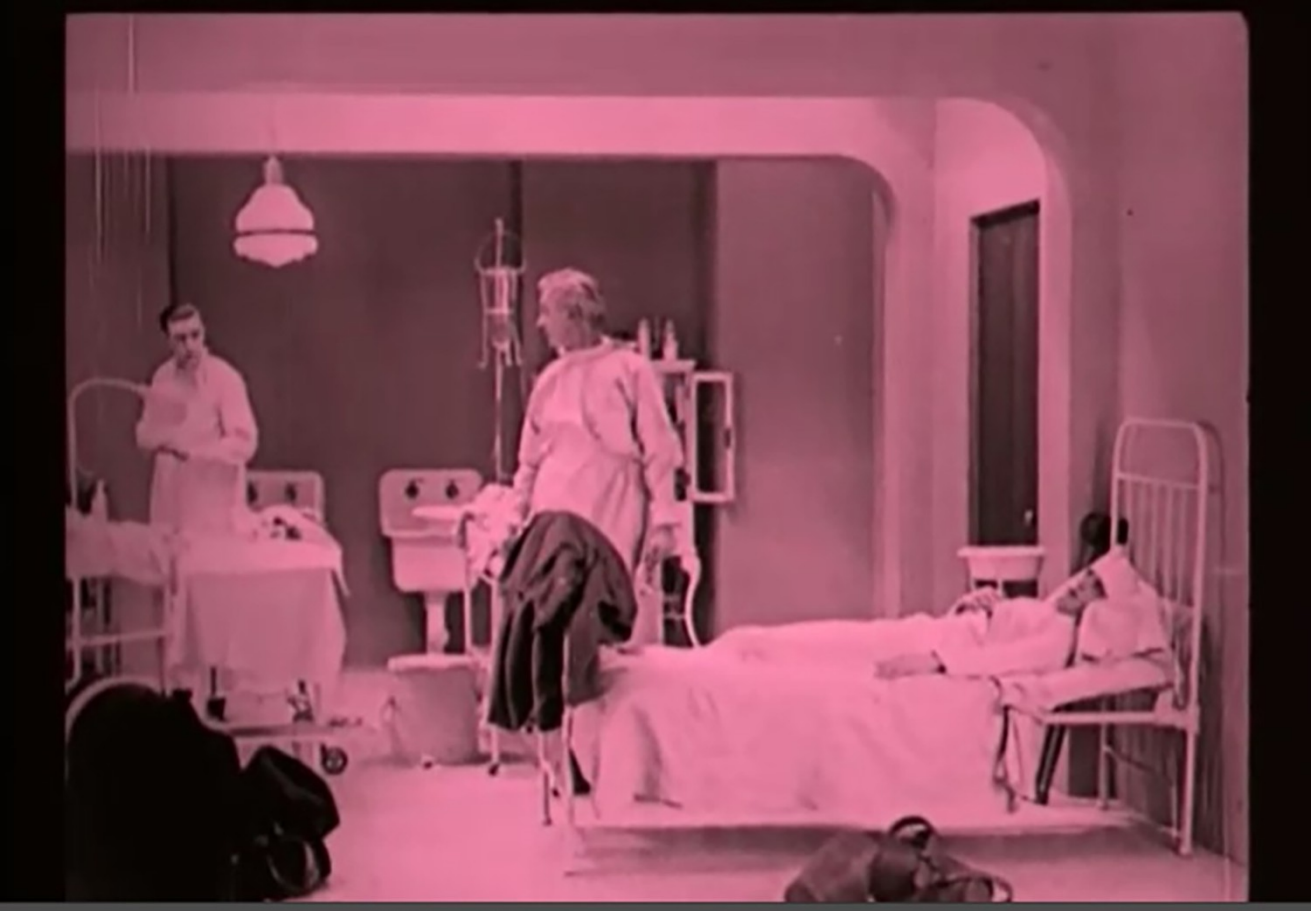
The Penalty (1920). The villain after the successful neurosurgical intervention.

Not so much monetary interest but carelessness marks the next surgeon from *The Penalty* (USA 1920, Wallace Worsley). The surgeon commits a grave error that causes his patient to lose both legs at a young age. From then on, the crippled man seeks revenge on his tormentor, turns into a criminal mastermind. He befriends the doctor’s daughter in order to carry out his vendetta. In the end, the doctor manages to save his daughter from the violent maniac: The gangster boss tries to force him to transplant the legs of a healthy person onto his torso. When the villain is out of consciousness, the heroic physician takes advantage of the situation and places the scalpel on the criminal’s forehead–with success. Thanks to the neurosurgical intervention (Fig 1), the obsession for revenge is eliminated and a curious but reconciliatory conclusion emerges.

Film example 3: *Der Gang in die Nacht* (Germany 1921, Friedrich Wilhelm Murnau). Dr. Eigil Börne is a famous surgeon and ophthalmologist, who values his career more than his private life. The doctor’s wife willingly restrains her own desires for the sake of her husband’s career. When the couple visits an opera house, the physician is called backstage because of a medical emergency. The supposed emergency turns out to be a minor ankle injury to the opera diva. The woman flirts with the doctor, but the latter rejects her for the time being. But at the follow-up visitation he loses his demeanor and begins an affair with her. He leaves his wife and goes on a trip with his mistress. Arriving at their destination, Börne, who has a resume of hundreds of surgeries, identifies a new professional challenge: He meets a blind village painter, whom he cures with a complicated operation. For the surgeon, this is a triumph with an unpleasant side effect, because the patient steals the doctor’s mistress. But when his rival’s eyesight fades again, the embittered physician imposes a horrible condition: He will operate on the painter again if the unfaithful mistress takes her own life. There is no happy ending: The woman commits suicide, the doctor sinks–disillusioned and abandoned–into his chair and the painter remains blind. Director Murnau, known for the expressionist classic *Nosferatu* (Germany 1922), created a visually compelling work of art. The mise en scène ranges from romantic compositions à la Arnold Böcklin or Casper David Friedrich to the rough shapes and contrasts of expressionism. Conrad Veidt, famous for the lead role in *Das Cabinet des Dr*. *Caligari* (Germany 1920, Robert Wiene), shines as the almost stereotypical spiritualized, mystical-looking painter. Although he takes another man’s wife, he is not portrayed in a negative way. The character of the doctor, on the other hand, is anything but favorably portrayed: he cheats on and leaves his wife, puts his career above his private happiness and uses his powerful medical position to drive his rival into suicide. He also appears to have little enthusiasm for his profession, as illustrated by a scene in which he snarls back "No thanks (…) duty" in response to a "thank you" from one of his patients. Why he became so embittered remains unanswered, but the ambitious pursuit of the surgical profession is repeatedly shown to be time-consuming and incompatible with a healthy family life. The doctor misinterprets his wife’s consideration for his time-consuming work as indifference and seeks diversion with the seemingly exciting opera singer. The further moral downfall–up to blackmailing suicide–seems to be a formality, a way out of the downward spiral is never indicated as an option. In the end, we are left with a disastrous portrayal of an ambitious surgeon, the profession itself and its ethics. When a world-class surgeon completely abandons professional ethics when facing a personal crisis, one question lingers: Do doctors have too much power? For Murnau’s film, this has to be confirmed. In summary: A profound, artistically outstanding work that raises uncomfortable questions about the surgical profession. The movie was long considered lost, incomplete, and even longer without intertitles, which explains its rather limited reception by film historians and primes it for a long overdue rediscovery [24].

**Fig 2:**
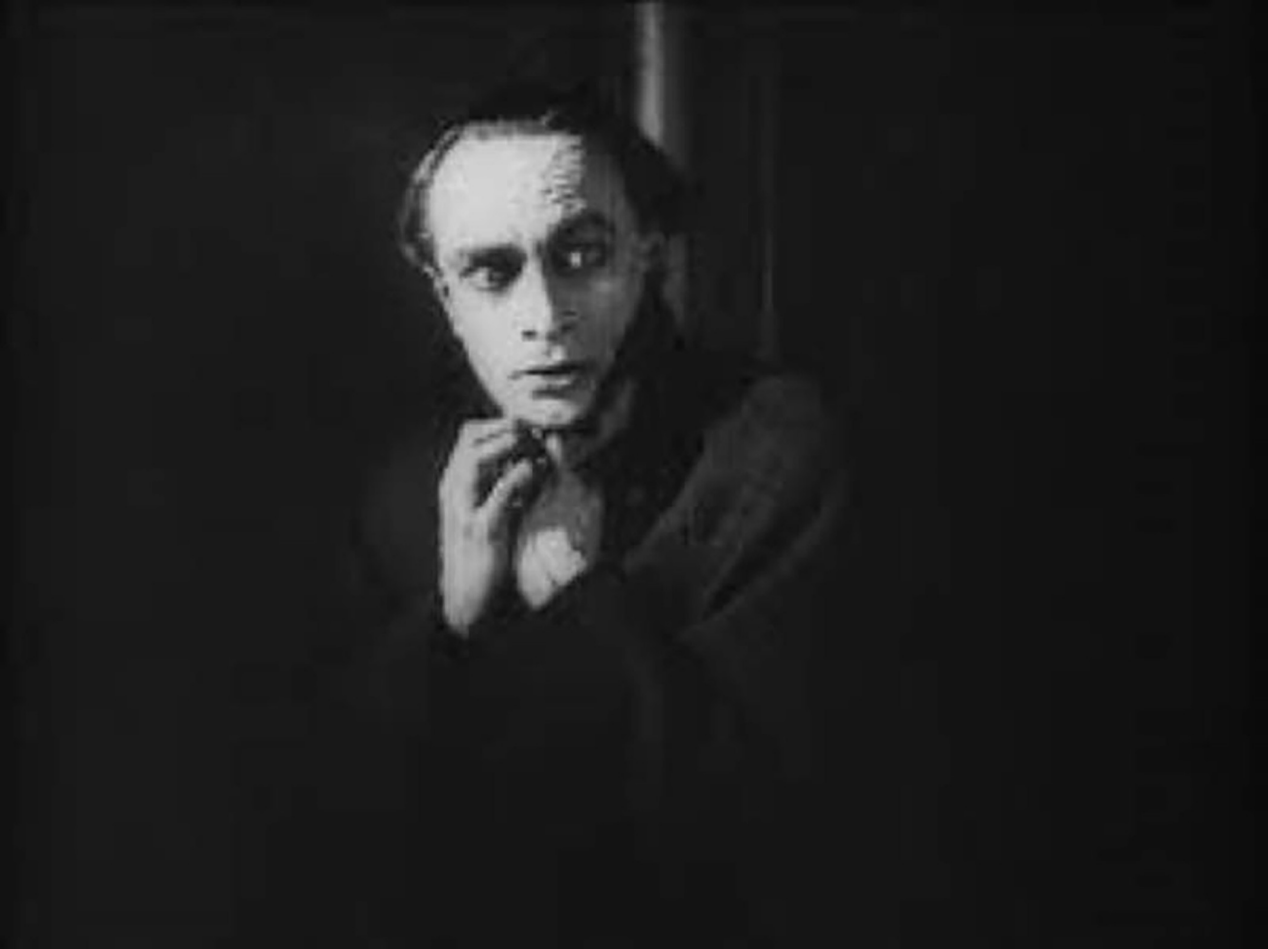
The hands of Orlac (1924). Orlac suffering from a paranoid episode.

We see Conrad Veidt again in the expressionistic *Orlac’s Hände* (Austria 1924, Robert Wiene), again in collaboration with the "Caligari creator" Robert Wiene. Veidt plays the world-famous pianist Orlac, who loses both hands in a train accident. Nevertheless, modern medicine is there to help, and two hands are transplanted onto the injured man. However, the patient suspects the donor was a murderer and suffers paranoid episodes (Fig 2), which the physician, against his better judgment, does not clear up–because he has his eyes on Orlac’s mistress. In the end, everything is clarified, but even this time the doctor appears shady as soon as personal (romantic) interests influence his actions. Certainly, the non-disclosure of the donor may be in conformity with modern donor protection laws, but here, we see a condition of the most severe suffering that is approved or deliberately maintained by the physician–a procedure that could lead to the loss of his license to practice medicine today [25]. Film historians even interpreted Veidt’s doctor figure as that of an aggressor, initiating the insanity symptoms actively rather than passively billing them: "slowly driven to madness" is the verdict there [26].

An even more sinister doctor character is embodied by Lon Chaney in *The Monster* (USA 1925, Roland West), which clearly stresses the cliché of the mad genius: The surgeon, Dr. Ziska, provokes serious car accidents on a secluded country road by mirror tricks. He takes his victims to an abandoned sanatorium that the insane man once ran. There, the maniac–"once a famous surgeon"–tries to perform a "most remarkable operation" on the casualties, which is supposed to make them immortal. However, the young detective Jennings finds out about the sadistic doctor’s plans and manages to stop him. This surgeon character is not only the purest version of the "mad doctor" stereotype, but the abandoned hospital also resembles a true horror cabinet–surgical megalomania as an integral element of horror!

### Optical disfigurement and peculiar vanities—plastic surgery as an exceptional subject

Considering the visual nature of the medium of film, it is hardly surprising that plastic surgery with its disfiguring pathologies was a particularly frequent subject of motion pictures. The earliest work from this category is *Musty’s Vacation* (USA 1917, Louis Myll), a slapstick comedy satirizing a man’s quest to look like a magazine cover model. A certain "Dr. A. Skin–Beauty Doctor" tries to fulfill his wish, but achieves with his procedure–clearly inspired by the special effects of Méliès–only a grotesque extension of the patient’s legs and head. The surgeon in the comedy *Minnie* (USA 1922, Marshall Neilan, Frank Urson) accomplishes his duty more appealingly. Minnie is by far the ugliest girl in the village. However, thanks to her inner beauty, she is able to gain the love of a reporter who allows her to have a surgical procedure. AThis makes her inner beauty visible to everyone, and the couple awaits a happy future [27]. Likewise, the Oscar-winning director Leo McCarey addressed the issue comically in *Mighty Like a Moose* (USA 1926). Here, a visually disfigured couple–with a monstrous overbite and a colossal nose–are operated on without each other’s knowledge. The success is so thorough that the two do not recognize each other and comic chaos emerges.

The impressive success of cosmetic surgery in drama films led to quite a few productions, as shown in Callé, 1994:

“Surgery changes people for the better in dramatic films. In *Defying Destiny*, a man’s face is burned while he is rescuing his sweetheart, and the surgeon removes the scars. In *The Man Who Married His Own Wife*, a sea captain’s face is disfigured when he saves a shipwrecked heiress. They marry, but he believes his wife does not love him. Surgery restores his face, but struggles with his earlier memories. In *One Way Street*, a British opera star is cast aside when her voice fails. She returned to a high-society life after her appearance was restored. In *fashion for women*, a fashion model retreats from public life and has a face lift. In *Gigolo*, a French soldier sustains facial injuries. He becomes a paid dancing partner in Nice after surgery and finds a mate. In *As a Man Lives*, the son of wealthy parents underwent plastic surgery, and his personality improved. An Apache he fights to seek a surgeon’s help to evade the police. In Scar Hanan, a rancher wrongly accused of cattle rustling saves a girl’s life. His scars are removed by her father, a plastic surgeon.” [28].

In addition to the purely aesthetic benefits, plastic surgery was utilized for more far-reaching purposes: The optical transformations were abused as camouflage, as in *The Hawk’s Nest* (USA 1928, Benjamin Christensen), in which a café owner postoperatively poses as a criminal; or to escape police pursuit, as in *Three Miles Up* (USA 1927, Bruce M. Mitchell), or even to provoke police prosecution, à la *Skin Deep* (USA 1922, Lambert Hillyer) [29]. An exceptional case is provided by *The Broken Mask* (USA 1928), in which the image of the plastic surgeon is more reminiscent of the dystopian doctor characters of his surgical screen colleagues from other fields: Dr. Gordon White has removed all facial scars from the dancer Perito, but when he falls in love with the artist’s wife, he tries to make the scars visible again. As punishment, the medic gets whipped for neglecting his professional ethics [30].

Film example 4: *Defying Destiny* (USA 1923, Louis Chaudet) deserves a closer look for many reasons: Jack rescues Beth from the blazing flames of a house fire, suffering disfiguring facial injuries in the process. But the tragedy also has advantages for the scarred man, because the father of the rescued girl is the manager of a bank, where Jack is now privileged to work. He earns handsomely, and manages to compensate for his cosmetic flaws with expensive sports cars and finally conquers Beth’s heart. Her father, however–since the disfigured one was only a tool to showcase his generosity–shudders at the thought of having a deformed man represent his family. Therefore, when embezzled money in the bank’s finances are discovered, it happens to be convenient for the patriarch to find a reason to fire Jack and publicly discredit him. Although the wrongly accused is cleared by a court, he is so "handicapped by the scarred face" that no one wants to employ him. After a year, he finally finds employment, but is seriously injured and hospitalized. However, the fall from a ladder does not cause any consequential damage. In the hospital he encounters a plastic surgeon–Dr. Gregory–who believes the reconstruction of the scarred face will bring him fame and fortune. If he could only correct these scars, the world would be at his feet–he even offers his patient $5,000, if he agrees to the operation. After a quick agreement, the surgeon fulfills his promise and the visually completely transformed Jack returns to the bank, disguised with a moustache. Everything pans out well for him: no one recognizes him, he gets back his old job, Beth falls in love with him (again) and even the disapproving father is impressed with the "newcomer". Jack takes advantage of the moment, reveals his identity, and says triumphantly grinning: "plastic surgery". A happy ending for all, the actual thief is exposed and even the questionably motivated doctor is now rewarded with glamour and glory. Were it not for the questionable incentives and (financial) gratuities to the patient, the film could serve as a promotional movie for the specialty. Expensive surgery was therefore a common motif of early "surgery cinema," but as *Defying Destiny* shows, it did not automatically imply defamation of the surgeon or its curative successes. It almost seems as if the viewer accepts the "surgical deus ex machina", even when displayed with a "minor character flaw" Financial motivation as a marginal character flaw–in a profession with such far-reaching responsibilities and consequences–still irritates as a questionable character approach.

In summary, early filmmakers portrayed plastic surgery in a largely positive light, providing an interesting contrast to the silver screen image of the talkie era, which–especially with a disastrous patient satisfaction rate of 7.7%–represents the specialty in a more than questionable manner [31].

### A second exceptional subject–The neurosurgeon

The depiction of neurosurgical interventions is rather uncommon in silent cinema, but one typical clinical condition–the craniocerebral trauma with all its devastating consequences–seems to have had a special attraction for audiences. By far the most popular of those consequences was amnesia, as shown in recent research:

“No fewer than 10 silent movies (before 1926) do so. In 1915, *Right of Way* was one of the first films to depict amnesia as the result of an assault, and the trigger for starting a new life. The same theme is used in *The Victory of Conscience* (1916) and has been constantly used through the decades until the modern era (…). In daily practice, most severe amnesia syndromes have a clear neurologic or psychiatric basis (…). Traumatic brain injury in cinema commonly causes a complete personality change (…) in one of the earliest cinematic examples, a roguish cad becomes a valued parish priest in *The Victory of Conscience* (1915). (…). Occasionally, traumatism results in a personality change for the worse, such as in *The Back Trail* in 1924, and *De Luxe Annie* in 1918. Such a scriptwriting template may find its origins in the cultural impact of the story of Phineas Gage, who famously had a dramatic and spectacular accident in 1848 while working on the railroad when an iron rod was driven through his head, destroying a large part of his left frontal lobe. (…) One of the most surprising clichés surrounding posttraumatic amnesia is the universal rule of “two is better than one” when it comes to head injury. In countless movies, amnesic characters regain full faculties, identity, and personality after a second blow to the head. From Tom Cat to Tarzan in *Tarzan The Tiger* (1922) [sic], another serious head injury can be totally and unexpectedly redeeming. (…) Even although it is occasionally depicted as the source of the amnesic syndrome, neurosurgery is usually considered a viable treatment option. In *De Luxe Annie* (1918), neurosurgery restores Annie’s memory (…).” [32]

In addition to this panorama of amnesic figures, other productions with correspondingly symptomatic patients can be noted: The German motion picture *Zweimal gelebt* (Germany 1914, Max Mack) portrays a doctor who takes advantage of his patient’s amnesic state and takes her away from her family and–without informing the kidnapped woman of her forgotten past–takes her as his wife. When the fraud is exposed, the deceived and humiliated woman commits suicide. The physician in *The Great White Trail* (USA 1917, Leopold Wharton, Theodore Wharton) is more successful, as two patients with post-traumatic, retrograde amnesia are cured by the confrontation with objects from their past.

However, traumatic brain injury provided more "canvas-symptoms" than just amnesia. Common were characters who became a psychotic danger to society after suffering head trauma. In *Hearts and Diamonds* (USA 1914, George D. Baker), a baseball thrown at a baseball player’s head is to blame for him wandering around disoriented and making the neighborhood unsafe. A year before *Zweimal gelebt*, Max Mack directed *Der Andere*, a classic German Film d’Art, which gives the traditional doppelganger motif a medical context: In the mold of the novella Strange Case of Dr Jekyll and Mr Hyde, a certain Dr Hallers commits crimes at night without being able to remember them during the day. Mack makes no secret of the etiology—in Haller’s case, a traumatic brain injury:

"As a result of a fall, overexertion or severe illness (…) create a double existence in man (…). The one knows nothing of the other. The pathological can commit acts in a kind of twilight state, of which the healthy part has not the slightest knowledge".

Theater celebrity Albert Bassermann plays the injured prosecutor Haller, whose "twilight state (…)" is cured by means of talking therapy–not without hinting at a relapse. The neurosurgical procedure itself can also be found in *The Penalty* (see above) as well as in *The Struggle* (USA 1916, John Ince), where the emergency operation is heroically performed on the open sea [33].

## Conclusions

The era overview illustrates that filmmakers often used surgery to create fear, suspense, or even plain horror. The doctor characters range from apotheotic saviors to lunatic monsters, resulting in a notably ambivalent "screen representation" of the field. The most trustworthy image among the specialties of surgery in early cinema was provided by the plastic surgeon, although his cinematographic reputation was greatly diminished in the sound film era. But why was the ambivalent surgeon so appealing to viewers? The answer can be found in the nature of the subject: Anesthetized (or suffering unimaginable pain), patients surrender to a complete loss of control, followed by the destruction of their body integrity by terrifying surgical instruments–with often unforeseeable consequences. By using surgery in their plots, filmmakers could create scenarios of unimaginable horror, without having to leave the boundaries of realism–especially at a time, when memories of (excruciating) procedures without anesthesia were still vivid in the minds of the audience. So vivid, that the term "surgical anxiety" was defined at the turn of the century [34]. However, this principle also works antithetically, because hardly any other medical discipline can achieve such healing successes as surgery, which makes the surgeon the prototypical deus ex machina, who can astoundingly prevent the worst kind of fates–especially the life-destroying optical disfigurements that make the actions of a plastic surgeon necessary. However, surgery truly enriched many excellent films by remarkable filmmakers such as Georges Méliès, F. W. Murnau, and Robert Wiene. Therefore, the analyzed film corpus–forty-one films from 1897 to 1929 –offers not only valuable primary sources for film and medical historians, but also culturally significant pieces of art that are worth (re-)discovering (Table 1). For more in-depth information, a table with the selection of films analyzed is attached.

**Table 1:** Chronological list of analyzed films.

Titel	Produktionsland	Erscheinungsjahr		Regisseur
Chirurgien américain	Frankreich	1897		Georges Méliès
Chirurgie fin de siècle	Frankreich	1900		Alice Guy
Une indigestion	Frankreich	1902		Georges Méliès
When Love Was Blind	USA	1911	Lucius J. Henderson	
Der Andere	Deutschland	1913	Max Mack	
The Back Trail	USA	1914	George Marshall, Clifford Smith	
Zweimal gelebt	Deutschland	1914	Max Mack	
Hearts and Diamonds	USA	1914	George D. Baker	
Right of Way	USA	1915	John W. Noble	
Das Tagebuch des Dr. Hart	Deutschland	1916	Paul Leni	
I’m Insured	USA	1916	Harry Palmer	
The Victory of Conscience	USA	1916	Frank Reicher, George Melford	
The Struggle	USA	1916	John Ince	
When Love Was Blind	USA	1917	Frederick Sullivan	
Musty’s Vacation	USA	1917	Louis Myll	
The Great White Trail	USA	1917	Leopold Wharton, Theodore Wharton	
Stella Maris	USA	1918	Marshall Neilan	
Good Night, Nurse!	USA	1918	Roscoe Arbuckle	
De Luxe Annie	USA	1918	Roland West	
Pollyanna	USA	1920	Paul Powell	
The Penalty	USA	1920	Wallace Worsley	
Der Gang in die Nacht	Deutschland	1921	F. W. Murnau	
The Affairs of Anatol	USA	1921	Cecil B. DeMille	
Back Pay	USA	1922	Frank Borzage	
Minnie	USA	1922	Marshall Neilan, Frank Urson	
The Man Who Married His Own Wife	USA	1922	Stuart Paton	
Skin Deep	USA	1922	Lambert Hillyer	
No Noise	USA	1923	Robert F. McGowan	
Defying Destiny	USA	1923	Louis Chaudet	
As a Man Lives	USA	1923	J. Searle Dawley	
Orlac’s Hände	Österreich	1924	Robert Wiene	
The Monster	USA	1925	Roland West	
One Way Street	USA	1925	John Francis Dillon	
Mighty Like a Moose	USA	1926	Leo McCarey	
Gigolo	USA	1926	William K. Howard	
Fashions for Women	USA	1927	Dorothy Arzner	
Three Miles Up	USA	1927	Bruce M. Mitchell	
Schmutziges Geld	Deutschland / Großbritannien	1928	Richard Eichberg	
The Hawk’s Nest	USA	1928	Benjamin Christensen	
The Broken Mask	USA	1928	James P. Hogan	
Tarzan The Tiger	USA	1929	Henry MacRae	

## Supporting information

S1 Table Chronological list of analyzed films. Representative selection of forty-one surgery films from the silent era.
(DOCX)
