# Supplementary material for: The two sides of the scalpel: The polarizing image of surgery in early cinema
Source: PLoS One. 2022 Dec 21;17(12):e0279422. doi: 10.1371/journal.pone.0279422 (PMC9770343; doi:10.1371/journal.pone.0279422)
Supplement: S1 Table — Representative selection of forty-one surgery films from the silent era. (DOCX) [file pone.0279422.s001.docx]

**Title**

**Country**

**Year**

**Director**

Chirurgien américain

France

1897

Georges Méliès

Chirurgie fin de siècle

France

1900

Alice Guy

Une

indigestion

France

1902

Georges Méliès

When Love Was Blind

USA

1911

Lucius J. Henderson

Der Andere

Germany

1913

Max Mack

The Back Trail

USA

1914

George Marshall,

Clifford Smith

Zweimal gelebt

Germany

1914

Max Mack

Hearts and Diamonds

USA

1914

George D. Baker

Right of Way

USA

1915

John W. Noble

Das Tagebuch des Dr.

Hart

Germany

1916

Paul Leni

I'm Insured

USA

1916

Harry Palmer

The Victory of

Conscience

USA

1916

Frank Reicher,

George

Melford

The Struggle

USA

1916

John Ince

When Love Was Blind

USA

1917

Frederick Sullivan

Musty's Vacation

USA

1917

Louis Myll

The Great White Trail

USA

1917

Leopold Wharton,

Theodore Wharton

Stella Maris

USA

1918

Marshall Neilan

Good Night, Nurse!

USA

1918

Roscoe

Arbuckle

De Luxe Annie

USA

1918

Roland West

Pollyanna

USA

1920

Paul Powell

The Penalty

USA

1920

Wallace Worsley

Der Gang in die Nacht

Germany

1921

F. W. Murnau

The Affairs of Anatol

USA

1921

Cecil B. DeMille

Back Pay

USA

1922

Frank Borzage

Minnie

USA

1922

Marshall Neilan, Frank

Urson

The Man Who Married

His Own Wife

USA

1922

Stuart Paton

Skin Deep

USA

1922

Lambert Hillyer

No Noise

USA

1923

Robert F. McGowan

Defying Destiny

USA

1923

Louis Chaudet

As a Man Lives

USA

1923

J. Searle

Dawley

Orlac’s Hände

Austria

1924

Robert Wiene

The Monster

Germany / UK

1925

Roland West

One Way Street

USA

1925

John Francis Dillon

Mighty Like a Moose

USA

1926

Leo McCarey

Gigolo

USA

1926

William K. Howard

Fashions for Women

USA

1927

Dorothy

Arzner

Three Miles Up

USA

1927

Bruce M. Mitchell

Schmutziges Geld

Germany / UK

1928

Richard Eichberg

The Hawk's Nest

USA

1928

Benjamin Christensen

The Broken Mask

USA

1928

James P. Hogan

Tarzan The Tiger

USA

1929

Henry MacRae
